# Dual Task Impairments in Vascular Dementia

**DOI:** 10.3233/BEN-2009-0252

**Published:** 2010-06-10

**Authors:** Kate Inasaridze, Jennifer A. Foley, Robert H. Logie, Sergio Della Sala

**Affiliations:** ^1^Tbilisi State Medical UniversityMemory ClinicTbilisiGeorgia; ^2^Human Cognitive NeuroscienceUniversity of EdinburghEdinburghUK; ^3^Centre for Cognitive Ageing and Cognitive EpidemiologyUniversity of EdinburghEdinburghUK

**Keywords:** Dual-tasking, vascular dementia, Alzheimer’s disease, memory performance

## Abstract

Several studies have shown that people with Alzheimer's disease (AD) demonstrate difficulties in doing two things at once or 'dual-tasking' and that this dual task impairment is insensitive to normal ageing, chronic depression or prodromal conditions like Mild Cognitive Impairment. It is not known, however, if this impairment is specific to AD, or also present in other dementias, such as vascular dementia (VaD). In this study 15 people with VaD, 25 healthy age-matched and 25 healthy young controls were assessed using a paper and pencil dual tasking paradigm and several measures of working and episodic memory. Age had no effect on dual task performance, but the VaD patients demonstrated a significant impairment in dual tasking ability. Performance on the memory measures was instead affected by age with a further deterioration in the VaD patients. Both dual tasking and memory ability were significantly correlated with disease severity, as assessed by the MMSE. These results indicate that performance on the dual task could be a specific indicator of pathological ageing.

## 1. Introduction

Dual tasking is the ability to perform two activities concurrently. The ability to dual task is thought to reflect the capacity to coordinate the concurrent activity of two independent cognitive systems [5,6,19].

Once performance on each individual task component is equated across participants, dual task performance is not affected by healthy ageing [6,8,16,20,34, 35]. Older people with chronic depression likewise do not have a difficulty with dual task demand, even when their performance on episodic memory tasks has been equated with that of AD patients [18].

On the other hand, it has been repeatedly shown that, in addition to the episodic memory impairment, people with AD display a striking impairment in dual tasking ability, when compared with healthy age-matched controls [3,6,10,15,19,23,24,27,28,35]. Moreover, this dual task deficit appears to become more pronounced with the development of the disease and increasing severity [2,3,10]. This dual task impairment does not appear to be present in Mild Cognitive Impairment (MCI) in general [11,12], although lower dual task performance in individuals with MCI has been found to be predictive of later conversion to AD [30]. Taken together, these findings suggest that the dual task impairment is specific to AD, and is not a marker for non-converting MCI, depression or healthy ageing.

This AD-specific dual task impairment cannot easily be explained by suggesting that dual task is simply more difficult than single tasks, and therefore more likely to be compromised by neurodegenerative disease. First, difficulty can refer to a wide variety of aspects of task demand, and cannot readily be determined in advance of observing performance, thereby making it a rather circular concept. Second, there is empirical evidence to suggest that AD patients are not particularly sensitive to manipulations of task demand of each individual component task. Logie et al. [19] examined the impact on AD patients and healthy older participants of varying the demand of single tasks compared with requiring

ISSN 0953-4180/09,10/$17.50 © 2009/2010 – IOS Press and the authors. All rights reserved concurrent performance of two tasks. They found that the AD group always displayed dual task impairment even when the demand of each task was set well below their individual span. However, the AD patients showed exactly the same sensitivity to increasing demand on a single task as did the controls. Moreover, the AD-specific dual task impairment is robust over practice [4,11] and different task combinations, including memory/motor tasks (e.g. [6]) and visual/verbal memory task combinations (e.g. [24]).

Although there is considerable evidence to suggest that the dual task impairment is specific to AD, and not present in normal ageing, non-converting MCI or chronic depression, it is not known if this impairment is present in other forms of dementia.

Vascular dementia (VaD) is the second most common form of dementia after AD [17]. The neuropsychological syndrome of VaD is characterised by attentional and executive dysfunction [13,32,33] with relatively preserved memory functioning [21,31], hence some have argued (e.g. [31]) that VaD should be primarily thought of as a dysexecutive disorder. Dual tasking has been considered to be an executive function [1,5,25], and thus it is possible that people with VaD will also demonstrate deficits in dual task coordination.

Only one study examining dual tasking in VaD is known to the authors. Graham et al. [13] compared 19 people with VaD, 19 people with AD and 19 healthy participants on a range of measures of cognitive functioning, including dual tasking. They found that the VaD patients performed significantly more poorly than healthy controls and the AD patients on the assessment of dual tasking ability. However, this comparison between VaD and AD may be an artefact of the patient recruitment strategy. The selection of VaD patients included only those who had substantial white matter pathology, vascular risk factors and a history of transient ischaemic attacks, and did not use the NINDS-AIREN (National Institute for Neurological Diseases and Stroke Association – Association International pour la Recherché et l'Enseignement en Neurosciences) criteria for probable vascular dementia. It is possible, therefore, that the VaD patients selected for this comparison consisted entirely of those with small vessel disease subtype.

McPherson and Cummings [22] discuss the various subtypes of vascular dementia and their characteristic patterns of cognitive impairment. They note that small vessel disease VaD subtype features considerable deficits in attention (including set shifting, verbal fluency and abstraction). Thus, the gross impairment in dual task coordination in the patients in the Graham et al. [13] study may indicate that this impairment is part of the neuropsychological syndrome specific to this subtype of vascular dementia, and not vascular dementia as a whole. Moreover, Graham et al. [13] examined dual tasking ability using an earlier version of the current paradigm [10], which had low reliability (0.36). In the study reported here, we used a newer version of the dual task paradigm, which has been found to have good test-retest reliability in both younger (0.83) and older adults (0.90) based on normative data from 128 healthy participants aged 17–75 years [16].

This aim of this study is to explore how people with VaD perform on a dual task assessment, when compared with healthy age-matched controls. In addition, all participants underwent a number of working and episodic memory tasks, to elucidate further the neuropsychological profile of VaD and the relationship between dual-tasking and working memory.

The experimental hypotheses were that the people with VaD will demonstrate (1) dual task impairment and (2) difficulties on the working and episodic memory assessments, when compared with healthy age-matched controls.

## 2. Method

### 2.1. Participants

Sixty-five people participated in this study. This consisted of 15 patients with VaD, 25 healthy older adults and 25 healthy younger adults.

The 15 VaD patients had been diagnosed in accordance with the NINDS-AIREN and ICD-10 criteria for probable vascular dementia. The patients had multi-infarct dementia, with heterogeneous patterns of cortical and subcortical involvement, and concomitant atrophy of other brain areas. The VaD patients (7 females, 46.7%) had a mean MMSE of 19.67 (SD = 4.64, range 11–25). They ranged in age from 47 to 75 years (mean = 65.60, SD = 8.64), and in formal education from 2 to 17 years (mean = 13.27, SD = 3.90). The mean duration of VaD was 3.30 years (SD = 2.51, range = 0.50–10.00 years).

The 25 healthy older participants were matched as closely as possible to the VaD group for age and education. The healthy older adults (14 females, 56.0%) had a mean MMSE of 29.04 (SD = 0.83, range 27–30). They ranged in age from 52 to 75 years (mean = 63.48,

SD = 6.94), and in formal education from 8 to 16 years (mean = 14.42, SD = 2.24).

The 25 healthy younger participants (14 females, 56.0%) had a mean MMSE of 29.78 (SD = 0.52, range = 28–30). They ranged in age from 19 to 25 years (mean = 20.80, SD = 1.80), and in formal education from 11 to 16 years (mean = 13.96, SD = 0.52).

### 2.2. Procedures

#### 2.2.1. Dual task assessment

Each participant completed the dual task assessment, which consisted of performing a digit recall and a tracking task separately and then simultaneously. Before commencing the digit recall task, digit span for each individual was established. Participants heard a list of digits at a rate of one per second. These digits had been digitally recorded and were played to the participants via a computer. Participants were then asked to repeat these digits back in the same order as they heard them. The initial sequence length was two digits and participants were presented with six sequences at each sequence length. If five out of the six sequences were recalled correctly, the digit sequence was lengthened by one digit. Digit span was taken as the maximum length at which the participant was able to recall five out of six digit sequences correctly. After the participant's span had been established, they heard sequences at their individual span length for immediate oral serial recall, and this was repeated for as many sequences as could be presented and recalled over a 120 second period. Therefore, the number of lists for each participant varied depending on the length of their digit span, and the performance measure was the proportion of digits recalled correctly.

The tracking task consisted of using a felt pen to draw a line through a series of black arrows arranged in a path around a sheet of A3 paper. Participants were given a shortened version for a practice trial, with only 35 arrows, to ensure that they understood the task demands. After this, the participant was presented with the full version comprising 373 arrows, and asked to start at one end of the path and draw a line through each successive arrow as quickly as they could for a period of 120 seconds. The performance measure was the number of arrows crossed by the pen.

In the dual task condition, participants were asked to perform the tracking task at the same time as listening to and repeating back the digit sequences they heard, for a further 120 seconds. The performance measures were the proportion of digits recalled accurately and the number of arrows crossed by the pen. Proportional performance in digit recall ($p$m) was calculated by measuring the change in digit recall between single ($m$single) and dual task ($m$dual) conditions, where $m$ is the proportion of digits recalled accurately, and using:

$$p_{\rm m} = \frac{100 - [(m_{\rm single} - m_{\rm dual}) \times 100]}{m_{\rm single}}$$

Proportional performance in tracking ($p$t) was calculated by measuring the change in tracking between single ($t$single) and dual task ($t$dual) conditions, where $t$ is the number of arrows drawn through, and using:

$$p_{\rm t} = \frac{100 - [(t_{\rm single} - t_{\rm dual}) \times 100]}{t_{\rm single}}$$

Proportional performance in both tasks overall ($\mu$) was calculated by using:

$$\mu = \frac{p_{\rm m} + p_{\rm t}}{2}$$

In addition, all participants completed several measures of working and episodic memory. These were:

#### 2.2.2. Working memory span task

This task was based on the method used by Baddeley et al. [7] to assess working memory span. On this test, participants heard a series of short sentences, half of which were true, e.g. 'Doctors have a profession', and half of which were false, e.g. 'Florida is a parent'. For each sentence, participants were asked to indicate whether it was true or false, and to remember the last word heard (e.g. parent). Initially, the participant heard three blocks of two sentences, was asked to verify each sentence immediately after its presentation by saying 'true' or 'false', and then at the end of each block, to recall the last word for each sentence, in any order. If, for two out of three blocks, the last words were recalled correctly, the number of sentences in each block increased by one. Working memory span was taken as the mean of the three longest sequences of sentences for which the participant was able to recall the last words on two out of three blocks, (e.g., for sequences of five sentences with an error in recall of the third sequence, span would be (5 + 5 + 4)/3 = 4.67.

#### 2.2.3. Verbal paired associates

Verbal Paired Associates (VPA) from the Wechsler Memory Scale (Third Edition) is a test assessing ability to make word associations. Participants are read eight word-pairs, such as 'rose – glass'. Participants are then read the first word and asked to recall its pair. Four trials are given of the same list, although the word-pairs are presented in different orders. Then, after around 30 minutes, this recall trial is again repeated.

Table 1
Dual task performance of digit recall, tracking and overall

| | Healthy younger Mean (SD) | Healthy older Mean (SD) | VaD Mean (SD) |
|---|---|---|---|
| Digit recall | 101.15 (6.50) | 103.27 (11.74) | 95.15 (8.38) |
| Tracking | 98.72 (9.56) | 98.78 (18.86) | 77.34 (23.07) |
| Overall | 99.94 (5.82) | 101.02 (12.08) | 88.48 (11.17) |

#### 2.2.4. *Word list learning*

This subtest from the Wechsler Memory Scale (Third Edition) requires participants to listen to a list of 12 words, presented over four trials. This list is then followed by one trial of a different list of words (an interference list), and next participants are asked to recall the 12 original words from the first list. After around 30 minutes, this recall trial is again repeated.

## 3. Results

### 3.1. *Analytical strategy*

Means and standard deviations were calculated for each of the variables. Normality of distribution was assessed using the Kolmogorov-Smirnov test and, if significant, by examining the $z$-scores for skewness and kurtosis. Homogeneity of variance was assessed using the Levene's test. Unless otherwise stated, all data met the assumptions of normality and homogeneity of variance.

### 3.2. *Participant characteristics*

An independent $t$-test revealed no significant difference in age between the VaD patients and healthy older participants [$t$ (38) $= -0.85, p = 0.40$].

A Kolmogorov-Smirnov test revealed that there was not a normal distribution of years of education in the three participant groups [$D$ (65) $= 0.21$, $p < 0.001$], with a significantly negatively skewed education distribution in both the older adults ($z$skewness $= -3.47$) and VaD patients ($z$skewness $= -3.12$), as well as a significantly leptokurtic distribution in the VaD patients ($z$kurtosis $= 3.80$). In addition, there was insufficient homogeneity of variance between the three groups in education [$F$ (2, 62) $= 4.20$, $p < 0.05$]. A Kruskall-Wallis test was used, therefore, to determine if there were any significant differences in education between the three groups. This revealed that there were none [$H$ (2) $= 2.06, p = 0.36$].

MMSE scores were not normally distributed in the three participant groups [$D$ (61) $= 0.32$, $p < 0.001$], with a significantly negatively skewed ($z$skewness $= -5.13$) and leptokurtic ($z$kurtosis $= 6.27$) distribution in the healthy controls. In addition, a Levene's test revealed that there was insufficient homogeneity of variance between the three participant groups [$F$ (2, 58) $=$ 33.40, $p < 0.001$]. A Kruskall-Wallis test revealed significant group differences in MMSE scores [$H$ (2) $=$ 42.69, $p < 0.001$] and post-hoc Mann Whitney tests revealed that the VaD patients had a significantly lower MMSE score than both the healthy young ($U =$ 0.00, $p < 0.001$, $r = -0.87$) and healthy older participants ($U = 0.00$, $p < 0.001$, $r = -0.83$), and with a significant difference between the two healthy groups ($U = 124.00$, $p < 0.05$, $r = -0.49$), after Bonferroni correction.

#### 3.2.1. *Dual task performance*

Group means and *SD*s of proportional performance in digit recall ($p$m), tracking ($p$t) and both tasks overall ($\mu$) are presented in Table 1.

There was a significant difference in dual-task performance overall [$F(2, 58) = 8.36, p < 0.05, \omega = 0.44$] and Bonferroni corrected post-hoc group comparisons revealed that the VaD patients performed significantly worse than the healthy younger ($p < 0.001$, $r = 0.84$) and older participants ($p < 0.05$, $r = 0.47$), with no significant difference between the two healthy groups ($p = 0.70$, $r = -0.06$).

Figure 1 shows a Receiver-Operating-Characteristic (ROC) curve, depicting the relative sensitivity and specificity of the overall dual task measure for the VaD group, when compared with the healthy older participants. The dotted diagonal line indicates the expected finding should the dual task measure provide zero discrimination, yielding an area under the curve of 50%. The figure illustrates that the measure is both sensitive and specific in discriminating between the VaD group and healthy older adults. The area under the curve is 0.79 (SE $=$ 0.08), which is significant ($p < 0.05$). Confidence intervals are 0.63 (lower bound) and 0.94 (upper bound). However, this analysis is limited by the small number of participants included.

Table 2
Performance on memory assessments

| | Healthy younger Mean (SD) | Healthy older Mean (SD) | VaD Mean (SD) |
|---|---|---|---|
| Working memory span | 3.06 (0.76) | 2.47 (0.70) | 1.47 (0.74) |
| Visual paired associates (immediate recall) | 21.28 (7.86) | 12.83 (7.87) | 2.27 (2.52) |
| Visual paired associates (delayed recall) | 6.16 (2.19) | 3.50 (2.83) | 0.53 (1.06) |
| Word list learning (immediate recall) | 38.64 (4.30) | 29.79 (7.15) | 13.40 (4.49) |
| Word list learning (delayed recall) | 9.68 (2.21) | 5.21 (2.93) | 0.40 (0.91) |

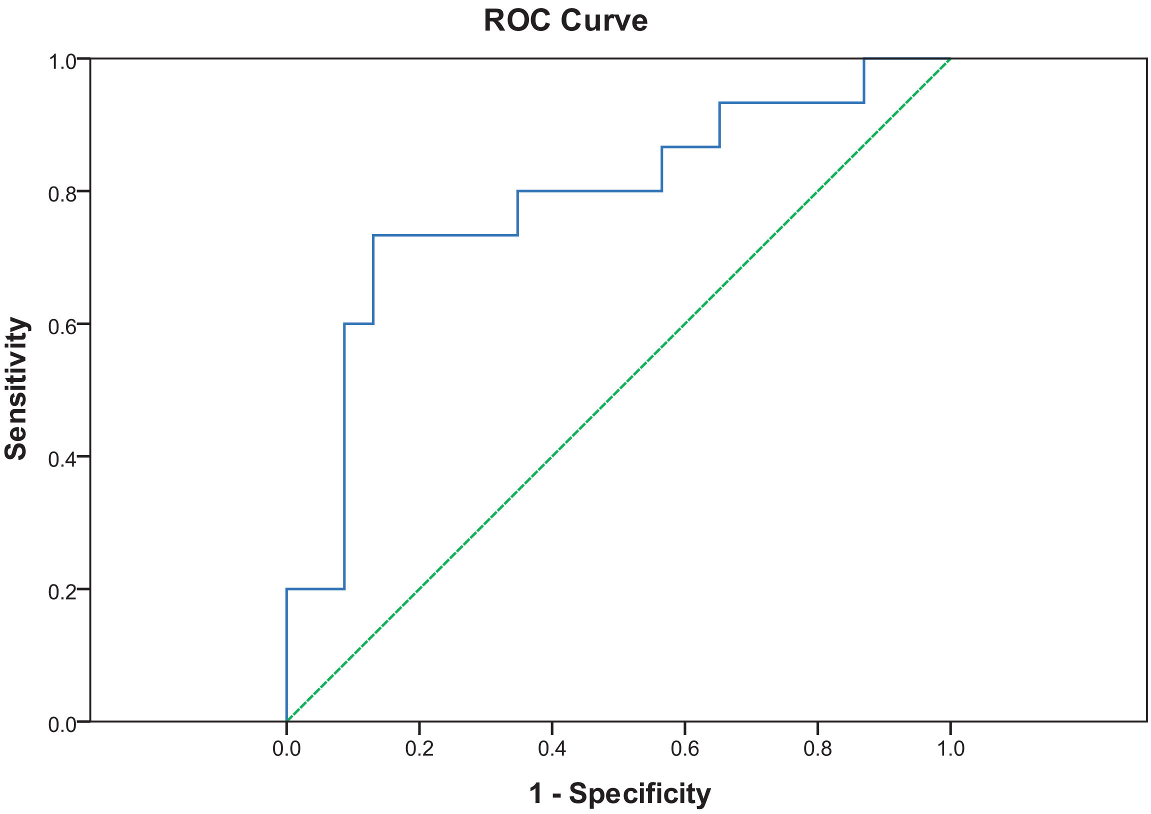

Fig. 1. ROC-Curve depicting sensitivity and specificity of the overall dual task measure. This curve compares the VaD group using the healthy older adults as a reference group (higher sensitivity scores indicate lower performance).

### 3.3. Relationship between dual task performance and MMSE score

There was a significant correlation between dual-task performance and MMSE score in the whole sample ($\rho = 0.43$, $p < 0.001$), but not when the sample is stratified by group (healthy younger: $\rho = -0.17$, $p = 0.43$; healthy older: $\rho = 0.34$, $p = 0.12$; VaD: $\rho = 0.45$, $p = 0.09$), although this could be due to the reduced sample size and in the healthy controls by the skewed distributions of the scores. There was also no correlation between dual task performance and duration of VaD ($r = -0.09$, $p = 0.75$).

### 3.4. Memory performance

Group means and SDs of memory tasks performance are presented in Table 2.

Performance on the working memory span measure was not normally distributed in the three groups [$D$ (64) $= 0.22$, $p < 0.001$]. Performance was significantly positively skewed and leptokurtic in both the healthy young ($z$skewness $= 5.28$; $z$kurtosis $= 9.93$) and healthy older participants ($z$skewness $= 6.01$; $z$kurtosis $= 11.41$). A Kruskal-Wallis test revealed that there was a significant difference in the working memory spans of the three participant groups

[$H(2) = 35.91$, $p < 0.001$]. Post-hoc Mann-Whitney tests revealed that VaD patients performed significantly more poorly than the healthy younger ($U = 4.50$, $p < 0.001$, $r = -0.82$) and older participants ($U = 49.50$, $p < 0.001$, $r = -0.64$), and the healthy older participants performed significantly more poorly than healthy younger participants ($U = 122.50$, $p < 0.001$, $r = -0.51$), when using Bonferroni correction.

One-way between-participants analyses of variance showed that there was a significant difference in the immediate recall version of both the Verbal Paired Associates [$F$ (2, 61) = 34.75, $p < 0.001$, $\omega = 0.71$] and the Word List Learning tests [$F$ (2, 61) = 95.90, $p < 0.001$, $\omega = 0.87$]. Bonferroni corrected post-hoc group comparisons found that there were significant differences between all groups. On the Verbal Paired Associates test, the VaD patients performed more poorly than healthy younger ($p < 0.001$, $r = 0.89$) and older participants ($p < 0.001$, $r = 0.75$), and the healthy older participants performed more poorly than the healthy younger participants ($p < 0.001$, $r = 0.48$). On the Word List learning test, the VaD patients performed more poorly than healthy younger ($p < 0.001$, $r = 0.94$) and older participants ($p < 0.001$, $r = 0.79$) and the healthy older participants performed more poorly than the healthy younger participants ($p < 0.001$, $r = 0.65$).

Performance on the delayed recall version was not normally distributed for either Verbal Paired Associates [$D$ (64) = 0.21, $p < 0.001$, $\omega = 0.68$] or Word List Learning [$D$ (64) = 0.15, $p < 0.001$, $\omega = 0.84$]. On the delayed version of the Verbal Paired Associates test, performance was significantly positively skewed in the healthy young participants ($z$skewness = 3.03) and positively skewed and leptokurtic in the VaD patients ($z$skewness = 4.84; $z$kurtosis = 7.93). A Kruskal-Wallis test revealed that there was a significant difference in delayed recall on this test [$H$ (2) = 31.16, $p < 0.001$]. Post-hoc Mann-Whitney tests revealed that VaD patients performed significantly more poorly than the healthy younger ($U = 66.00$, $p < 0.016$, $r = -0.81$) and older participants ($U = 49.50$, $p < 0.001$, $r = -0.54$), and the healthy older participants performed significantly more poorly than healthy younger participants ($U = 137.50$, $p < 0.016$, $r = -0.47$), when using Bonferroni correction.

On the delayed version of the Word List Learning, performance was significantly positively skewed and leptokurtic in the VaD patients ($z$skewness = 3.98; $z$kurtosis = 4.16). A Kruskal-Wallis test revealed that there was a significant difference in delayed recall on this test [$H$ (2) = 47.73, $p < 0.001$]. Post-hoc Mann-Whitney tests revealed that VaD patients performed significantly more poorly than the healthy younger ($U = 0.00$, $p < 0.001$, $r = -0.84$) and older participants ($U = 30.00$, $p < 0.001$, $r = -0.71$), and the healthy older participants performed significantly more poorly than healthy younger participants ($U = 69.50$, $p < 0.016$, $r = -0.66$), when using Bonferroni correction.

## 4. Discussion

Several studies have reported that dual task performance is affected by normal ageing [14], and a number of neurological conditions, including MCI [26]. However, when the two tasks are individually adjusted to equate single task performance across groups, dual task performance is not affected by normal ageing [6,8,16, 20,34,35], non-converting MCI [12,30] or chronic depression [18]. When the dual-task has been titrated taking into consideration the performance of each individual single task, it has been consistently shown that people with AD demonstrate impairment in dual task coordination, when compared with healthy controls [3, 6,10,15,19,23,24,27,28,35].

The present study examined whether this dual task deficit is specific to AD or is present in other types of dementia also. People with VaD displayed significant dual task impairment, when compared with healthy controls. This suggests that dual task performance is impaired in different forms of dementia, but not in normal ageing, once care is taken to equate individual performance on the component single tasks. In addition, dual task ability is significantly correlated with disease severity, as measured by the MMSE.

As expected this study also found that VaD patients display impairment in memory when compared to healthy controls. Interestingly though, the healthy older adults also exhibited deficits in all the memory measures used here. This confirms that memory abilities deteriorate with age and that they deteriorate further in VaD. These findings contradict the suggestion that there is a selective sparing of memory in VaD [21, 31], but do replicate the findings of Graham et al. [13], who also found that VaD patients performed worse than healthy controls on a number of working and episodic memory measures.

Memory assessments are therefore sensitive, but not specific to pathological ageing, as memory performance in the current study, as well as in previous literature (e.g. [9]) was affected by normal ageing as well, and had been found impaired in chronic depression [36] and MCI [29]. In contrast, dual task assessment appears to be specific to dementia and not sensitive to these other disorders. The specificity of the dual task impairment indicates that there is some form of cognitive function responsible for coordinating the activity of two independent cognitive systems that is available in the healthy brain, but which becomes impaired with the onset of dementia.

In addition, this paper-and-pencil version of the dual task assessment is inexpensive, easily transportable and usable by primary health care staff with a minimal amount of training, making it suitable for clinical use. Thus, dual task may be a useful complement to measures of episodic memory in the assessment and in the differential diagnosis of dementia.

## Acknowledgements

This work has been partly funded by the grant AS-90-2007 awarded by the Alzheimer's Society to Sergio Della Sala and Robert H. Logie. Jennifer A. Foley is the appointed Research Fellow on this grant. Kate Inasaridze was funded by a Royal Society bilateral travel grant to Sergio Della Sala, and an INTAS Fellowship.
